# Drug resistance bacterial isolates in inpatients at Cocody University Hospital, Abidjan, Côte d’Ivoire in 2014

**DOI:** 10.1186/2047-2994-4-S1-P167

**Published:** 2015-06-16

**Authors:** KN Adele

**Affiliations:** 1 Bacteriology Virology, Pasteur Institute of Cote d’Ivoire, Abidjan, Côte d’Ivoire

## Introduction

Nosocomial infections by the emergence of antibiotic-resistant bacteria are also a real health problem in limited-resource countries. The high frequency of these infections goes along with increased consumption of antibiotics.

## Objectives

The aim of this study was to determine drug resistance rate of indicator bacteria isolated from nosocomial infections in inpatients.

## Methods

Hospital bass cross-sectional study was conducted on 299 isolates from inpatients of Cocody University hospital from january to december 2014. Bacteriological culture and examination was done following standard microbiological techniques. Drug resistance test was performed by disk diffusion methods against classes of antimicrobials. The data was analysed for descriptive statistics using EPI Info version 6.2 and Microsoft Excel.

## Results

Of the total of 299 isolates, strains were respectively from surgical (30.4%), pediatrics (18.7%), medicine (17.7%), neurology (14%), pulmonology (11.4%) and intensive center unit (7%). Enterobacteriaceae were represented by 60.2%, including *Escherichia coli* (24.4%) and *Klebsiella pneumoniae* (19.1%). A total of 92 (51.1%) enterobacteria producing extended spectrum beta-lactamase (ESBL). ESBL have been commonly isolated in pediatric, surgical and intensive care unit, especially in the urine. The rate of resistance to ciprofloxacin were 68.3% for Enterobacteriaceae. About 62 isolates of Staphylococci (20.7%), 45 were *Staphylococcus aureus* (72.6%) and 17.8% were resistant to methicillin (MRSA). Multidrug resistance rate was 4.4%. MRSA were common in surgery especially in suppurations.

Nearly, 10% of *Pseudomonas aeruginosa* were resistance to ceftazidime (CRPA). These strains were isolated in intensive care and pediatrics units.

## Conclusion

This study revealed that the rate of drug resistance was high for ESBL. These trends need to be monitored regularly. These data should be taken into account in the strategies against nosocomial infections.

## Disclosure of interest

None declared.

